# Emotional Dysregulation and Altered Reward Processing in Self-Harm

**DOI:** 10.1192/j.eurpsy.2022.1083

**Published:** 2022-09-01

**Authors:** E. Yavuz, M. Di Simplicio, R. Rodrigues

**Affiliations:** 1 Imperial College London, Psychiatry, NN, United Kingdom; 2 Imperial College London, Brain Sciences, London, United Kingdom

**Keywords:** self-harm, psychiatry, emotion regulation, reward processing

## Abstract

**Introduction:**

Self-Harm (SH) is any act of self-injury carried out by somebody irrespective of motivation. SH most commonly functions to relieve negative affect (NA). Tentative evidence suggests reward processing is altered in SH. NA may trigger reward hypersensitivity and therefore SH. Whether NA influences reward processing in SH remains unclear.

**Objectives:**

To investigate whether self-harmers differ in motivation to obtain SH stimuli than healthy controls (HCs) following NA induction.

**Hypothesis:**

After NA induction, SH participants will have a significantly shorter reaction latency (RL) and greater reaction accuracy (RA) than HCs in the SH condition of the Incentive Delay (ID) task.

**Methods:**

16-25-year-old SH (n=35) and HC (n=20) participants were recruited online and underwent the Trier Social Stress Test, to induce NA, followed by the ID task, where participants were cued to respond to a target as quickly as possible. On responding, an image of either a SH act (SH Condition), people socializing (Social Condition) or money (Monetary Condition) appeared. Each condition included control trials showing a neutral image. RA was the percentage of trials responded to within the target’s presentation time. RL was the time (seconds) between target appearance and participants’ response.

**Results:**

There was no significant main effect of group, condition or group x condition interaction for RL. There was a significant main effect of condition (p < 0.05) but not of group nor a group x condition interaction for RA.

**Conclusions:**

Reward processing did not differ in the SH group compared to HCs post-NA induction. Future studies could investigate reward processing in longitudinal and larger SH samples.

**Disclosure:**

No significant relationships.

